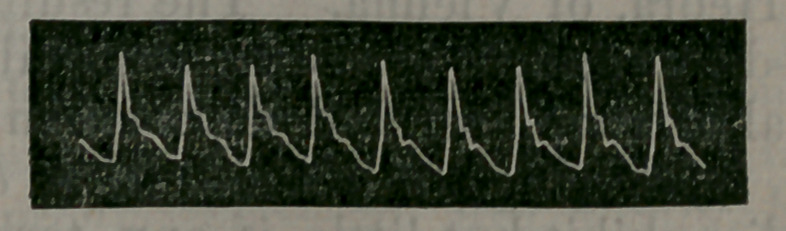# Pond’s American Sphygmograph

**Published:** 1878-09-20

**Authors:** Frank Woodbury

**Affiliations:** Philadelphia


### POND'S AMERICAN SPHYGMOGRAPH.

(Read before the Philadelphia County Medical Society.)

BY FRANK WOODBURY, M.D., of Philadelphia.

Having, by request of Professor DaCosta, and under his direction, made some trial, in the wards of the Pennsylvania Hospital, of the sphygmograph invented by Dr. E. A. Pond, of Rutland, Vermont, I wish to exhibit this instrument to the Philadelphia County Medical Society, and to communicate the results of our observations. The invention is protected by a patent issued by the United States to Dr. Pond in 1875. This instrument was first brought to the notice of the profession in November, 1875, when it was exhibited by Dr. Pond, at the meeting of the Suffolk District Medical Society, an account of which will be found reported in the Boston *Medical and Surgical Journal*, of December 23, 1875. Since that period, the inventor and his son, Dr. Wallace R. Pond, have shown it at a number of medical meetings, but not in the form in which it is now seen. The form originally was simply that of a sphygmoscope, which idea was naturally followed by the conception of the addition of a recording apparatus to convert it into a sphygmograph. Many improvements were gradually added until the instrument assumed its present shape, under which it was first presented before Dr. Stella's section of the International Medical Congress, held in this city during the centennial year.

This sketch of the history of the invention is given because much of the same principle of construction is adopted in the sphygmograph of Dr. Keyt, of Cincinnati, who published a description of his instrument in January, 1876, in the New York *Medical Journal*, volume xxiii., page 26, in an article entitled "The New Sphygmograph, or Instrument Adapted as a Sphygmograph, Sphygmometer, Cardiogroph, Cardiometer, and to Other Uses." It is unfortunate for Dr. Pond that no full description of his instrument had appeared anywhere previous to this publication by Dr. Keyt, for, although the principle of construction was undoubtedly verbally explained by Dr. Pond, at the meeting of the Suffolk District Medical Society, the invention has been credited to Dr. Keyt by writers, among others, Dr. F. G. Smith, in the American edition of Dr. Carpenter's work on Physiology, published in 1876. In a private letter from Dr. E. A. Pond, dated Rutland, April 24, 1877, he says: "I have been five years at work on it, and completed it about two years ago, and have been using it myself, to perfect it, before bringing it out, and am just cemmencing to bring it to the notice of physicians," which explains his delay in publication, evidently desiring it to assume its permanent form before publishing it fully.

The instrument differs from that of

Marey in transmitting the impulse of the artery not immediately to the lever, but indirectly, through the motion communicated to a column of water and a glass float, which finally moves the recording pen. The construction and character of the new sphygmograph, as seen in the instrument, and as shown in the cut, are readily understood. The main portion consists essentially of an upper and a lower glass tube. The lower tube, containing fluid and having a rubber diaphragm stretched on the lower end, is, in use, the part applied to the pulse. The upper and smaller tube fits the larger and lower tube by means of a packing on its inferior end, thus moving freely in and out, and determining, at desire, the height of the fluid in the small part of the tube. Inside the small part of the tube is a free float, made of glass, which floats according to the height of the fluid, and obeys any movement of the fluid, or any vibrations from the rubber cap on the lower end of the instrument. A pendulous-jointed needle clasps on the upper part of the tube. A watch movement is also attached to the tube to move the slide upon which the trace is to be made. A holder clasping the wrist, fastened by means of a sliding bolt, retains the instrument in place over the artery. A dial may be added, which shows the amount of pressure used.

An extra tube, having a larger bottom, is prepared for cardiographic traces.

The application of this instrument is simple, and, indeed, it may, after a little practice, be used off-hand—that is, holding it as you would a pen; apply the rubber diaphragm to the artery, vein or heart; use the requisite pressure to bring the float against the arm of the needle; place the free end of the needle on the slide; smoke very slightly a slide of mica, glass or paper; put the end between the rollers, start the movement, it will run through, and the needle will trace the pulse on it. Photographers' varnish will fix the trace so it can be handled. In using the holder, place the pressure dial on the lower end of the bolt, and have the blades of the holder between the dial and the nut, and slide it into place, the bolt fitting the slot in the end of the holder. Care must be taken to see that it is applied exactly over the artery.

There is also attached a ruler, so that the slide can be divided into millimetres while it is moving along, as seen in one of the specimens exhibited. These are two millimetres apart. Dr. Pond states that he has also used the needle reversed to move down on the slide, and can provide the case with both kinds of needles. I am also informed that these instruments are now made with a governor on the watch movement, so that it can be run at any rate of speed desired.

Professor DaCosta has been much pleased with the tracings made in this manner, and says that some of them are the finest he has ever seen.

I have found that by the aid of Pond's sphygmograph, I have obtained far better tracings of the radial pulse than I ever succeeded in getting with Marey's instrument. Owing to peculiarities in the instrument, and delicacy of construction, it requires considerable practice in order to become acquainted with its capabilities, and to gain skill in using it, but the results are so much better than with any inferior instrument that the experimenter feels repaid for his trouble immediately on comparing the tracings.

The cut shows a normal radial tracing obtained from the pulse of Dr. Pond.

Among the several tracings herewith submitted may be seen some taken from cases of aortic and mitral disease, typhoid and scarlet fevers, and from children at different ages. Of the numerous sphygmograms I have made, these are selected for their clearness and characteristic features, some of them being almost typical of the condition that supplied them. While the sphygmograph has not thus far obtained for itself the position of a *sine qua non* in the diagnosis of any particular affection, yet I feel warranted, at least, in saying, in view of these specimens, that it offers corroborative testimony which is capable of strongly confirming impressions arising from the usual methods of physical examination.

I think that the members of this society, who examine the tracings presented, will appreciate the advantages of the American sphygmograph, which, although not entirely free from fault, is, perhaps, better adapted to the purpose than any other instrument with which I am acquainted.—*Reporter*, *Phila.*

## Figures and Tables

**Figure f1:**
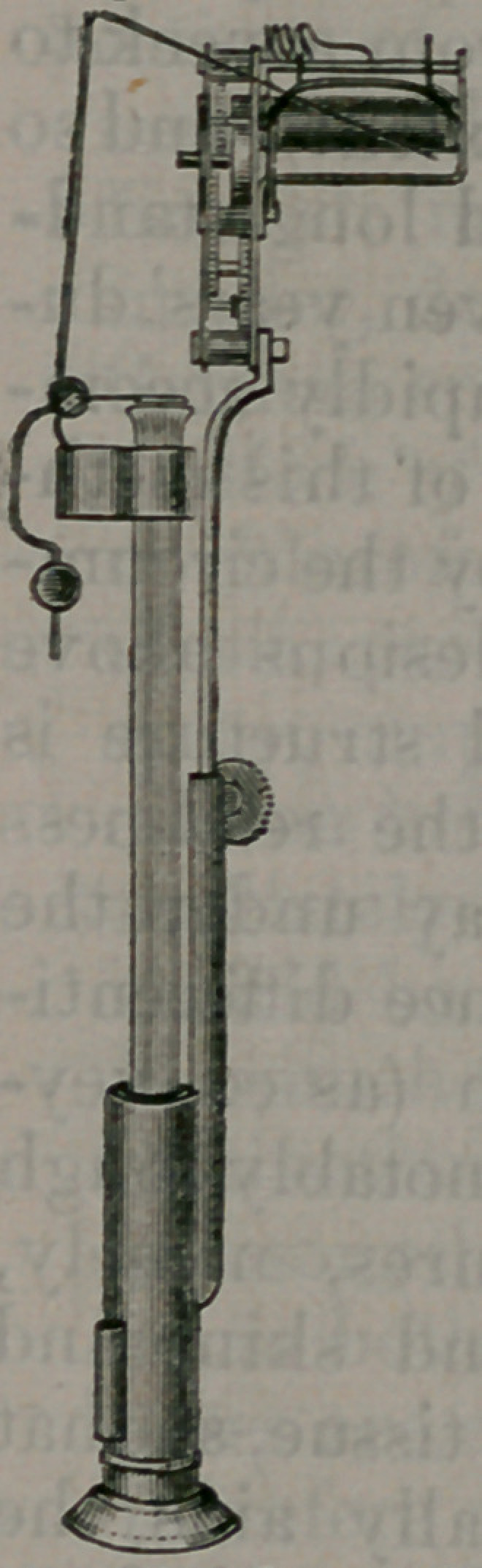


**Figure f2:**